# Case Report: Patient With Lung Adenocarcinoma With *ALK-HLA-DRB1* Rearrangement Shows Impressive Progression-Free Survival After Sequential Crizotinib and Ceritinib Treatment

**DOI:** 10.3389/fonc.2022.762338

**Published:** 2022-02-25

**Authors:** Peng Gao, Kangning Tang, Yuqiu Hao, Wei Li, Xuejiao Lv, Dapeng Li, Yuxi Jia

**Affiliations:** ^1^ Department of Respiratory and Critical Care Medicine, Second Hospital of Jilin University, Changchun, China; ^2^ Department of Respiratory and Critical Care Medicine, The Affiliated Hospital of Jilin Medical College, Jilin, China; ^3^ Department of Orthopedics Application Demonstration Center of Precision Medicine Molecular Diagnosis, the Second Hospital of Jilin University, Changchun, China

**Keywords:** ALK-HLA-DRB1 fusion variant, non-small cell lung cancer, ceritinib 3, crizotinib, ceritinib

## Abstract

The anaplastic lymphoma kinase (ALK) gene rearrangement is a driving mutation that underlies about 5-6% of non-small cell lung cancer (NSCLC) cases. Lung cancers that are ALK gene rearrangement-positive can be effectively treated with ALK inhibitors. However, the response of patients with rarer ALK gene rearrangements to ALK inhibitors remains unknown. Herein, we described a case of lung adenocarcinoma carrying ALK-HLA-DRB1 fusion in a 48-year-old nonsmoking woman. A similar case of ALK-HLA-DRB1 rearrangement in NSCLC has not been described previously neither in NSCLC nor in other disease. The patient achieved a progression-free survival of 18 months after sequential therapy consisting of crizotinib and then ceritinib during the follow-up. These findings provide basis for the application of ALK inhibitors in patients carrying the rare ALK-HLA-DRB1 fusion.

## Introduction

One of the most common human malignancies and the leading cause of cancer-related deaths in the world is non-small cell lung cancer (NSCLC) (1). Symptoms occur late in the disease as a result of which most lung cancer cases are diagnosed at an advanced stage (2). Thus, the majority of patients with NSCLC lose the chance for curative surgery (3). In recent years, the introduction of several new and more effective targeted therapies has extended the survival of many of these patients (3).

The Next-generation sequencing (NGS) allows sequencing of a high number of nucleotides in a short time frame and has been widely implemented in oncology practice (4). It has moved into the clinics with the aim of sequencing long and complex genes and/or multiple genes tumor sample, in order to identify driver and/or targetable alterations to guide treatment decisions (4, 5). There are three types of anaplastic lymphoma kinase (ALK) gene mutations, including mutation, amplification, and rearrangement/fusion with other genes (5). ALK-rearrangement is the most form of mutations (5). A chromosomal rearrangement involving ALK has been estimated in about 5-6% of patients with NSCLC (6), who tend to be young and never- or light-smokers (7). Such cases are associated with a high incidence of brain metastases (8). Better understanding of molecular pathways, gained *via* next-generation sequencing (NGS) analysis, has led to novel targeted therapies in NSCLC (9). Tyrosine kinase inhibitors (TKIs) of ALK (i.e., ALK-TKIs), including crizotinib, ceritinib, and alectinib, have dramatically improved the prognosis of patients with NSCLC (10). When administration of ALK-TKIs is considered for the management of NSCLC, crizotinib is a first-line treatment (11). Ceritinib is a second-generation TKI that has been approved for brain metastasis of NSCLC involving ALK mutations (12).

Here, we describe a case of advanced lung adenocarcinoma harboring a rare ALK-HLA-DRB1 (major histocompatibility complex, class II, DR beta 1) gene fusion. The patient achieved a rapid and remarkable response after crizotinib treatment. For economic reasons, 6 months later the patient was switched to receiving oral ceritinib, and she responded well. As of this writing, the progression-free survival of this patient is more than 18 months.

## Case Report

**Figure 1:**
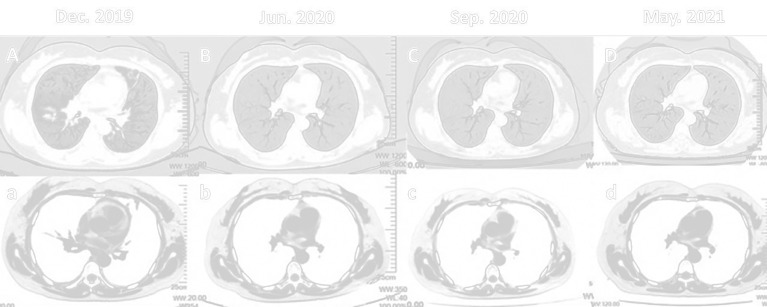
Dynamic chest computed tomography scans of the patient during treatment. Multiple nodules and patches in both lungs with blue arrow. **(A)** At initial diagnosis. **(B)** Six-months after initiation of crizotinib, remarkable reduction of multiple nodules and patches in both lungs, and improvement of mediastinal lymphadenopathy. **(C)** At 3 months of ceritinib treatment. **(D)** At 11 months after ceritinib treatment.

**Figure 2:**
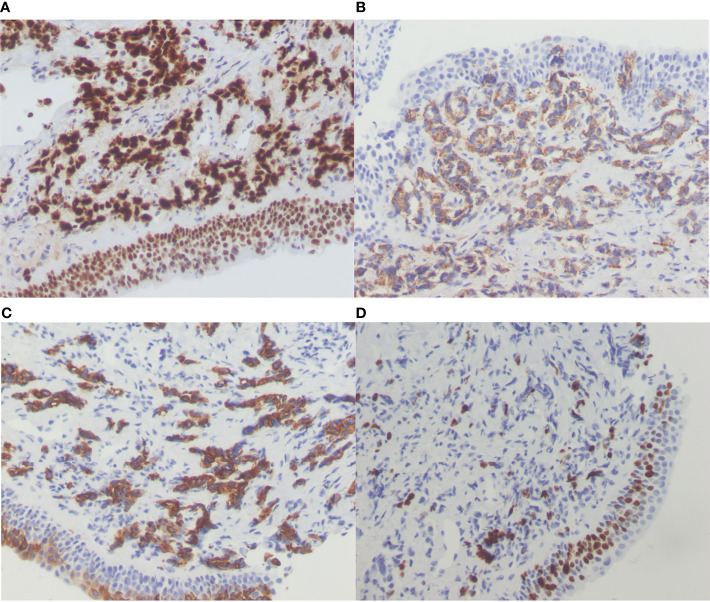
Histological findings. Immunohistochemistry was positive for **(A)** TTF-1 (200×); **(B)** Napsin A (200×); **(C)** CK7 (200×); and **(D)** Ki67 (200×).

On December 22, 2019, a 48-year-old Chinese woman presented to Second Hospital of Jilin University (Jilin, China) with a 4-month history of dry cough and progressive expiratory dyspnea. She was never a smoker. Chest computed tomography images displayed multiple nodules and patches in both lungs, mediastinal lymphadenopathy, and hydropericardium (**Figure 1**). Ultrasonography revealed enlargement of multiple lymph nodes at the left neck. Pathological examination of the bilateral supraclavicular lymph nodes and nodules in the right lower lobe by percutaneous needle biopsy revealed an adenocarcinoma of lung origin. Immunohistochemistry analysis revealed the positive presence of TTF-1, NapsinA, and CK7; and no signs of TG, P40, Syn, or CD56 (**Figure 2**). Multiple nodules and patches in both lungs and hydropericardium confirmed stage IVA lung adenocarcinoma, with a lost chance of surgery.

**Figure 3:**
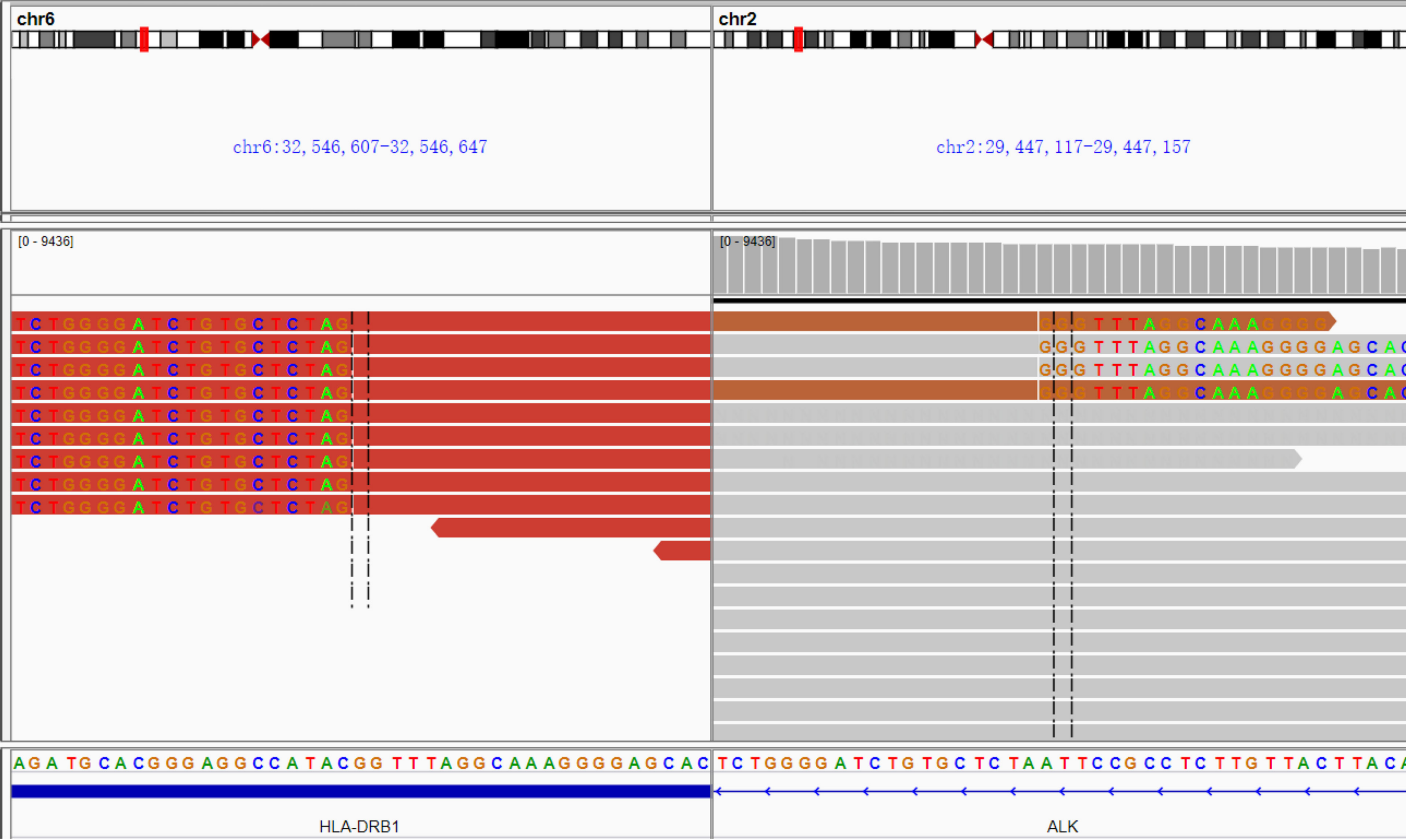
Screenshot of the Integrative Genomics Viewer from the next-generation sequencing fusion assay showing abundant supporting RNA reads with an ALK-HLA-DRB1 fusion, which consisted of exon 20 of the ALK gene and exon 8 of the HLA-DRB1 gene. The plasma ALK fusion abundance was 1.9%. Detection organization: Nanjing Shihe Jiyin Biotech Inc Laboratory; Detection platform: Illumina Hiseq/MiSeqDx; Reference genome: GRCh37/hg19.

Since the patient had no smoking history, we decided to investigate genetic alterations as potential targets, *via* NGS. The NGS of the serum sample revealed a gene rearrangement between exon 20 of the ALK proto-oncogene and exon 8 of the HLA-DRB1 gene, but signs were negative for MET, KRAS, MAP2K1, NF1, NRAS, PIK3CA, PTEN, BRAF, EGFR, and ERBB2. The ALK-HLA-DRB1 gene fusion, which retains the kinase domain of ALK (**Figure 3**), is presumed to lead to oncogenic activation of ALK. Based on this, the patient was administered the first-generation ALK-TKI crizotinib (250 mg, twice daily) from January to June 2020. She achieved a rapid response in both lungs and pericardium, which was maintained for 6 months (**Figure 1**). Clinical symptoms of dyspnea and cough were improved. There were no clinical or radiological signs of cerebral involvement or crizotinib resistance. The patient’s treatment was switched to ceritinib (600 mg per day) for economic reasons in July 2020. Follow-up imaging in September 2020 revealed an inspiring radiographic response, with decrease in the size of mediastinal lymph nodes and no new metastasis (**Figure 1**).

As of this writing, from the start of crizotinib administration, progression-free survival is more than 18 months. This case merits further follow-up and provides valuable information on the response to ceritinib of patients with NSCLC with ALK-HLA-DRB1 genetic fusion.

## Discussion

Anaplastic lymphoma kinase (ALK), discovered in 1994, is a transmembrane receptor tyrosine kinase that have been identified in several types of cancer, including anaplastic large cell lymphoma(ALCL) (13), inflammatory myofibroblastic tumor (14), and non-small-cell lung cancer(NSCLC) (5). More than 19 different ALK fusion partners have been described in NSCLC, including EML4, KIF5B, KLC1, and TPR (5). However, novel partner genes for ALK fusion and their clinical significance are not fully defined and HLA-DRB1 has not been reported as a fusion partner of ALK.

Human leukocyte antigen (HLA), located on chromosome 6, encodes genes that are crucial in the immune response to pathogens (15). There have been 4 described cases of HLA-DRB1-MET gene rearrangement in lung adenocarcinoma (16–19). Davies et al. reported the first case of HLA-DRB1-MET gene rearrangement who was detected a previously undescribed fusion of HLA-DRB1 exon 5 (NM_002124) to MET exon 15 (NM_000245) (18). And two cases of lung adenocarcinoma being driven by a HLA-DRB1-MET gene rearrangement with an excellent response to crizotinib (16, 18). However, to our best knowledge, there is no report of an ALK-HLA-DRB1 fusion variant in any cancer. The present case was a lung adenocarcinoma with an ALK-HLA-DRB1 fusion, which consisted of exon 20 of the ALK gene and exon 8 of the HLA-DRB1 gene. This is the first report of ALK-HLA-DRB1(A20:H8) as a gene fusion variant in NSCLC that is sensitive to crizotinib and ceritinib treatment.

ALK inhibitors are the standard initial therapy for NSCLC harboring an ALK fusion, and they provide rapid, profound, and durable responses (20). To understand better the appropriate application of ALK-TKIs, the sensitivity of a rare ALK-HLA-DRB1 fusion variant to ALK-TKIs should be explored.

In general, ALK-TKIs as a first-line treatment for ALK-positive NSCLC have extended progression-free survival, relative to pemetrexed plus platinum complexes (21). In the present case, the patient was treated with first-line crizotinib, at 250 mg twice daily, and achieved a partial response. However, after 6 months of administration of crizotinib, and without the development of crizotinib resistance or brain metastases, due to economic reasons the patient was switched to receive oral ceritinib and achieved progression-free survival for more than 18 months.

A preclinical evaluation showed that ceritinib, a second-generation ALK-TKI, is a more potent ALK inhibitor than crizotinib, penetrates the blood-brain barrier, and shows clinical responses in patients with crizotinib-resistant disease (22). In patients with ALK-positive NSCLC, brain metastases are frequent, occurring in approximately 60% to 90% of cases (23). Brain metastasis is a devastating complication of advanced lung adenocarcinoma, and is associated with rapid deterioration of performance (24). In previously reported cases, when switching to second-generation ALK-TKIs, crizotinib resistance or CNS metastases were common (25). Yet, our patient received ceritinib without development of crizotinib resistance or brain metastasis. Based on her progression-free survival of more than 18 months, we speculate that second generation ALK-TKIs such as ceritinib, given after crizotinib, may help prevent or delay brain metastasis and ALK-TKI resistance in ALK-positive NSCLC. That is, appropriate sequential treatment with first- and second-generation ALK-TKIs could lead to a good prognosis in NSCLC that involves a ALK-HLA-DRB1 fusion.

However, the best timing of drug replacement in sequential therapy in advanced-stage disease, with ceritinib as the initial drug, warrants further investigation. If a good response is not closely associated with the therapy, we can guess that the ALK-HLA-DRB fusion variant is a subtype of NSCLC with a good prognosis. In the study (26), strong HLA-DR-positive expression on cancer cells was significantly related to better prognosis and was an independent factor for better long-term survival for colorectal cancer patients, although the precise mechanism remains to be elucidated. HLA molecules play a critical role in engaging an antigen specific immune response acting, T cell priming and clonal expansion (26). We speculated that strong HLA-DR antigen expression in cancer cells may be associated with better prognosis of NSCLC patients by activating local immune response. It will be meaningful to accumulate, collect, and publish clinical practice data to clarify these issues.

## Conclusion

The fusion of ALK to HLA-DRB1 in any disease has not been reported previously. ALK-HLA-DRB1 gene fusion may be a class of actionable alteration in NSCLC. We provide evidence that patient with lung adenocarcinoma with ALK-HLA-DRB1 rearrangement shows impressive progression-free survival after sequential crizotinib and ceritinib treatment.

## Data Availability Statement

The original contributions presented in the study are included in the article/supplementary material. Further inquiries can be directed to the corresponding authors.

## Ethics Statement

Written informed consent was obtained from the individual(s) for the publication of any potentially identifiable images or data included in this article.

## Author Contributions

KT and YH collected the data, reviewed the literature, and drafted the manuscript. WL and XL participated in collecting the data and edited the pictures. YJ participated in writing-review and editing. DL provided image data and helped edit the pictures. PG made an important contribution to the diagnosis and treatment plan of the patient and revised the final version of the manuscript. All authors read and approved the final manuscript.

## Conflict of Interest

The authors declare that the research was conducted in the absence of any commercial or financial relationships that could be construed as a potential conflict of interest.

## Publisher’s Note

All claims expressed in this article are solely those of the authors and do not necessarily represent those of their affiliated organizations, or those of the publisher, the editors and the reviewers. Any product that may be evaluated in this article, or claim that may be made by its manufacturer, is not guaranteed or endorsed by the publisher.
